# Emerging Perspectives on Adverse Childhood Experiences and Brain Cancer Immunotherapy

**DOI:** 10.3390/cancers18121882

**Published:** 2026-06-09

**Authors:** John W. Figg, Julian Mark, Caretia J. Washington, Anna Fusco, Maegan A. Cremer, Scott A. Cohen, Stephan Quintin, Deidre B. Pereira, Ashley P. Ghiaseddin

**Affiliations:** 1 Preston A. Wells Jr. Center for Brain Tumor Therapy, Lillian S. Wells Department of Neurosurgery, University of Florida, Gainesville, FL 32611, USA; jwfigg@ufl.edu; 2 MD/PhD Program, College of Medicine, University of Florida, Gainesville, FL 32610, USAcaretia.washingt@ufl.edu (C.J.W.);; 3 J. Crayton Pruitt Family Department of Biomedical Engineering, University of Florida, Gainesville, FL 32611, USA; 4 Department of Neuroscience, College of Medicine, University of Florida, Gainesville, FL 32610, USA; 5 Center for Translational Research in Neurodegenerative Diseases, College of Medicine, University of Florida, Gainesville, FL 32610, USA; 6 Department of Clinical and Health Psychology, University of Florida, Gainesville, FL 32611, USA

**Keywords:** adverse childhood experiences/events, immunotherapy, brain cancer, neuroimmune

## Abstract

Adverse childhood events (ACEs) represent a major public health burden in the United States, as exposure to ACEs increases the risk of mental health disorders, cardiovascular disease, obesity, and cancer and can greatly affect quality of life. Although the effects of ACEs on physiology are complex, significant evidence suggests that ACEs may affect the immune system later in life. As the field of immunotherapy gains prominence in cancer treatment, it becomes imperative to explore how ACEs may impact cancer immunotherapy. Brain cancer, especially glioblastoma multiforme (GBM), has a very poor prognosis and remains a challenging disease to treat. Immunotherapy approaches are being actively investigated. To address this, we aim to raise awareness of the potential connection between ACEs, brain cancer, and cancer immunotherapy. Through a review of the current biomedical literature, we synthesized current findings into a commentary on how early-life adversity may influence the immune response within the central nervous system. It is the intention of this commentary to generate hypothesis-driven research within the nascent and emerging field of neuro-psycho-immunology in neuro-oncology. Given that the relationship between ACEs and brain cancer immunotherapy remains vastly understudied, this commentary has significant potential to generate insightful discussions among clinicians and scientists to guide further clinical and basic research towards anticipating which patients stand to receive the most benefit from immunotherapy.

## Simple Summary

Adverse childhood events (ACES) such as violence, abuse, and household dysfunction are highly prevalent in the United States with at least 60% of adults having experienced a minimum of 1 ACE. Both the direct bodily effects of chronic stress and high-risk behaviors associated with ACES are known to increase cancer risk in adulthood. Immunotherapies harness the patient’s immune system to fight cancer. However, several of the biomarkers for predicting immunotherapy success are known to be dysregulated in adults with a history of ACEs. Given the rapid progression of glioblastoma (median survival 10 months), it is imperative that patients rapidly receive the most effective therapy for their tumor. In this commentary, the authors describe the need to characterize the relationship between ACEs and immunotherapy response in glioblastoma with the long-term goal optimizing precision medicine in the context of ACE exposures.

## 1. Introduction

**Figure 1:**
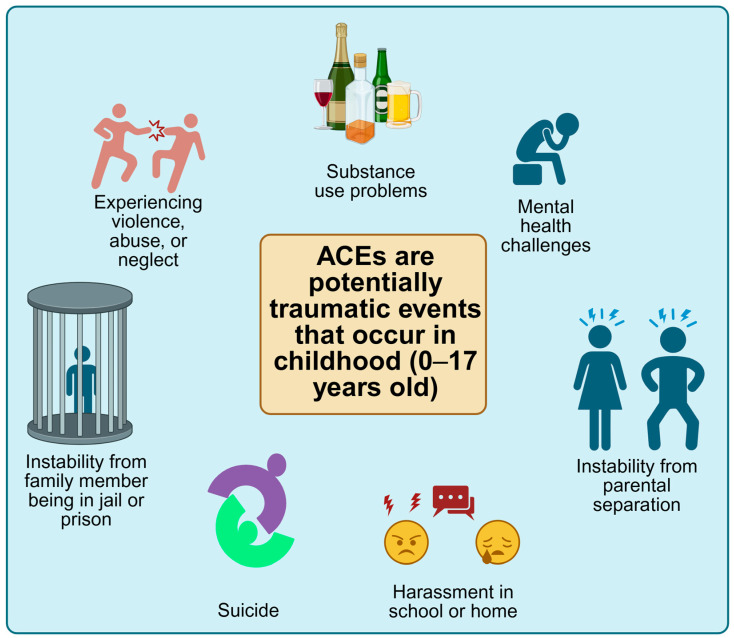
Common adverse childhood experiences that have been documented in biomedical literature.

Adverse childhood events (ACEs) are potentially traumatic events occurring before the age of 18 that include exposure to violence, abuse, and household dysfunction as described in Figure 1 [1,2]. According to the US Centers for Disease Control, ACEs are highly prevalent with approximately 64% of adults have experienced at least one ACE, while nearly 1 in 6 adults have experienced four or more ACEs [3]. ACE burden is not distributed equally across populations and disproportionately affects socially and economically vulnerable groups [4].

A substantial body of literature demonstrates that cumulative ACE exposure is associated with substantial adverse health outcomes across the lifespan. Compared to individuals without ACE exposure, those reporting four or more ACEs are more likely to experience poor self-rated health, cardiovascular and respiratory disease, accelerated epigenetic aging and increased cancer incidence in adulthood [5,6,7,8,9,10]. Higher ACE scores have also been associated with increased odds of having any psychiatric diagnosis, diabetes, coronary heart disease, and chronic pain syndromes [11,12,13,14,15,16]. More recent epidemiologic studies have reported associations between ACE exposure and increased incidence of melanoma and lung cancer, reduced adherence to cancer screening recommendations, and greater psychiatric morbidity among cancer survivors relative to individuals with no ACE exposures [17,18,19,20]. Parallel findings in preclinical models further support a biologically relevant role for early life stress in cancer pathogenesis, as exposure to stress during development has been shown to enhance carcinogenesis and accelerate tumor growth [21,22].

However, much of the current literature linking ACEs to cancer biology derives from observational studies and mechanistic work conducted with animal models outside of neuro-oncology. In many malignancies, disentangling the biologic consequences of ACE exposure from behavioral and socioeconomic confounders remains difficult because ACEs are also associated with smoking, alcohol use, substance use, reduced healthcare access, and chronic psychosocial stress [23,24,25,26,27,28,29,30,31,32,33].

Primary central nervous system tumors may provide a useful context in which to examine potential biologic effects of chronic stress exposure with fewer traditional behavioral confounders. Between 2018 and 2022, the average annual incidence of brain and other CNS tumors was 26.05 per 100,000 population [33,34,35,36]. Brain tumor incidence is not strongly associated with smoking or alcohol exposure, potentially allowing clearer examination of host immune and neuroendocrine factors [31,32,33,34,35,36]. Nevertheless, direct evidence linking ACE exposure to glioblastoma (GBM) development or therapeutic response remains limited. Accordingly, this commentary does not propose a causal relationship between ACEs and GBM outcomes. Rather, we discuss how chronic stress biology associated with ACE exposure may conceptually intersect with established mechanisms of immune dysfunction relevant to neuro-oncology. Because ACE exposure has been linked to chronic inflammatory pathways, neuroendocrine dysregulation and immune aging, it is plausible that ACE-associated stress biology could act as an additional host-level modifier of immune responsiveness. This hypothesis remains speculative in GBM and requires direct validation.

GBM remains the most common primary malignant brain tumor in adults and continues to carry a poor prognosis despite advances in surgery, radiation, and systemic therapy, with a median survival of approximately 10 months and a 2-year survival rate of only 6.7% [31,32,33,34,35,36,37,38]. One major contributor to these poor outcomes is the limited effectiveness of immunotherapeutic strategies in GBM compared with other solid tumors. Immune checkpoint inhibitors and other immunotherapies have transformed treatment paradigms of several malignancies including melanoma and non-small cell lung cancer (NSCLC) [35,36,37,38]. In GBM, clinical benefit following ICI is limited in part by an immunosuppressive tumor microenvironment, intratumoral heterogeneity, low neo-antigen burden, T cell sequestration, corticosteroid exposure and myeloid-dominant immunosuppression [39,40]. These tumor intrinsic and micro environmental factors are likely drivers of treatment resistance.

In this commentary, we review evidence linking ACE exposure to long-term immune alterations and discuss how these biologic changes may conceptually intersect with mechanisms of immunotherapy resistance in GBM, specifically over three broad domains: inflammatory priming, neuroendocrine dysregulation, and immune senescence. Throughout, we distinguish between findings established in psychoneuroimmunology and broader oncology literature alongside hypotheses that remain untested in neuro-oncology.

Collectively, these clinical and experimental findings suggest that psychosocial stressors during critical developmental windows may contribute mechanistically to cancer susceptibility later in life. Consistent with this hypothesis, multiple meta-analyses have demonstrated a significant association between ACE exposure and increased risk of adult cancer diagnosis [5,23,27].

## 2. ACES and the Immune System

### 2.1. Inflammatory Priming and Chronic Immune Activation

One of the most consistently reported biologic correlates of ACE exposure is persistent low-grade inflammatory activation [41]. Higher ACE burden has been associated with elevated circulating inflammatory markers, including C-reactive protein, interleukin-6 and tumor necrosis factor-alpha (TNF-α), as well as altered transcriptional profiles involving inflammatory signaling [41,42,43,44,45,46,47,48,49,50,51,52].

Several studies suggest that childhood adversity may re-calibrate immune responsiveness during critical developmental periods. For example, Schwaiger et al. demonstrated altered monocyte transcriptional responses to acute stress among individuals with histories of early life adversity [52]. The authors found that participants who faced greater early life adversity had greater activation of pro-inflammatory transcription factors including AP-1 and GATA in response to acute stress [52]. Similarly, childhood maltreatment has been associated with exaggerated IL-6 responses following acute psychosocial stress paradigms. History of bullying in childhood is associated with significantly increased plasma C-reactive protein (CRP) levels in adulthood, and this relationship is dose-dependent with the severity of exposure [40,41,42,43,44,45,46,47,48,49,50,51,52,53].

Parallel findings have been observed in animal models. Early life social deprivation in rhesus macaques and rodent stress paradigms has been associated with persistent inflammatory transcriptomic changes and altered immune regulation later in life [54]. While these studies support biologic plausibility, caution is warranted when extrapolating animal stress models to human malignancy. Most available evidence demonstrates associations with chronic inflammatory signaling rather than direct evidence of carcinogenesis or impaired tumor immunity. More recently, animal models with stress paradigms showed increased oxidative stress and evidence of psychopathology following stressful stimuli and may create a state conducive to chronic disease manifestation through elevations in acute phase reactants [55]. Oxidative stress responses in humans are also associated with childhood adversity. Adolescents experiencing four or more ACEs had significantly higher levels of F2t-isoprostanes in urine, a marker of oxidative stress [56]. However, clinical studies remain limited by their heterogenous design and inconclusive outcomes. Despite these limitations, oxidative stress and ACE scores were positively correlated with psychopathology such as major depressive disorder and type I bipolar disorder [56]. Prior mentioned literature on stress exposure and associations with acute phase reactants like CRP suggests concordance between animal models and humans, and thus similar inflammatory profiles following ACEs in adulthood.

Importantly, inflammatory dysregulation associated with ACE exposure is unlikely to be unique to ACEs specifically and may instead reflect broader chronic stress or acute stress. Chronic psychosocial stress, mood disorders, socioeconomic disadvantages and social isolation have all been associated with overlapping inflammatory phenotypes. Consequently, it remains difficult to determine whether observed changes are attributable specifically to childhood adversity or to cumulative lifelong stress exposure. This distinction is particularly important in neuro-oncology. Although inflammatory cytokines such as IL-6 have been associated with immunotherapy resistance in melanoma and NSCLC, there is currently no direct evidence demonstrating that ACE-associated inflammatory signatures independently predict immunotherapy outcomes in GBM. Rather, these observations provide a conceptual framework suggesting that chronic inflammatory states associated with early life adversity could therapeutically contribute to diminished immune responsiveness.

### 2.2. Neuroendocrine Dysregulation and Adrenergic Signaling

A second mechanistic domain involves chronic neuroendocrine dysregulation. Long-term activation of stress response systems, particularly the hypothalamic–pituitary–adrenal (HPA) axis and sympathetic nervous system, has been proposed as one pathway linking psychosocial adversity to immune dysfunction. The “conserved transcriptional response to adversity” (CTRA) model described by Cole and colleagues provides one framework for understanding this relationship [57]. In this model, chronic stress exposure is associated with increased adrenergic signaling and transcriptional programs favoring inflammatory gene expression while suppressing antiviral and adaptive immune responses [57]. This signaling, mediated by norepinephrine, engages intracellular cAMP/PKA pathways and downstream transcription factors such as CREB, NF-κB, and AP-1. The net effect is a shift toward pro-inflammatory gene expression with suppression of antiviral responses [57]. Experimental studies in humans further support a causal role for β-adrenergic signaling in stress-induced immune activation. Acute psychosocial stress increases inflammatory gene expression and cytokine production. These effects are significantly attenuated by β-adrenergic blockade with propranolol, indicating direct sympathetic regulation of immune transcriptional responses [58]. Complementing these observations, Haskó et al. demonstrate that β-adrenergic receptor stimulation directly suppresses IL-12 production in vivo [59]. This effect is reversible with propranolol and occurs independently of IL-10 signaling. These results highlight a catecholamine-mediated mechanism that constrains Th1-type immune responses during heightened sympathetic activity.

Importantly, early life stress appears to calibrate this system during critical developmental windows. In rhesus macaques, experimentally induced early social adversity produces long-lasting changes in immune gene regulation that mirror the CTRA profile [54,57]. This finding suggests early embedding of an adrenergic-mediated inflammatory bias. Parallel work in rodent models shows that early life stress alters the expression of adrenergic receptors and downstream signaling molecules, including α2A-adrenergic receptors and components of the cAMP/PKA pathway [60]. These findings indicate that stress can directly reprogram noradrenergic signaling architecture. At the systems level, these changes reflect increased sympathetic tone and repeated norepinephrine exposure. Together, they chronically bias leukocyte transcriptional activity toward inflammation. Collectively, these findings support a model in which ACEs induce durable alterations in adrenergic signaling. These alterations act as a key intermediary between early environmental adversity and long-term immune dysfunction, providing a biologically plausible pathway linking psychosocial stress to inflammatory disease risk.

Additional work suggests that catecholaminergic signaling can suppress IL-12 production and alter Th1 immune responses [59]. Animal studies further suggest that early life adversity may produce durable changes in adrenergic signaling architecture and stress responsiveness [59]. These findings support the possibility that ACE exposure may establish persistent neuroimmune phenotypes extending into adulthood. Given that GBM-mediated remodeling of neural circuits is associated with worse overall survival, future studies should investigate the impact of ACE-dysregulated neuroendocrine pathways in the CNS on GBM resistance [61,62]. It is conceivable that a neuroendocrine circuit or pathway could become dysregulated by an ACE exposure and mediate either gliomagenesis or treatment resistance [63,64,65]. However, direct translation of these observations is presently speculative, and current evidence has not identified an ACE-related adrenergic signaling pathway that alters immunotherapy responsiveness to GBM. Instead, these findings could suggest a biologically plausible pathway through which chronic stress biology could interact with host immune regulation. Taken together, dysregulation of neuroendocrine systems is well-supported within the psychoneuroimmunology framework, whereas its relevance to GBM immunotherapy remains hypothetical and incompletely studied.

### 2.3. Immune Senescence and Biologic Aging

**Figure 2:**
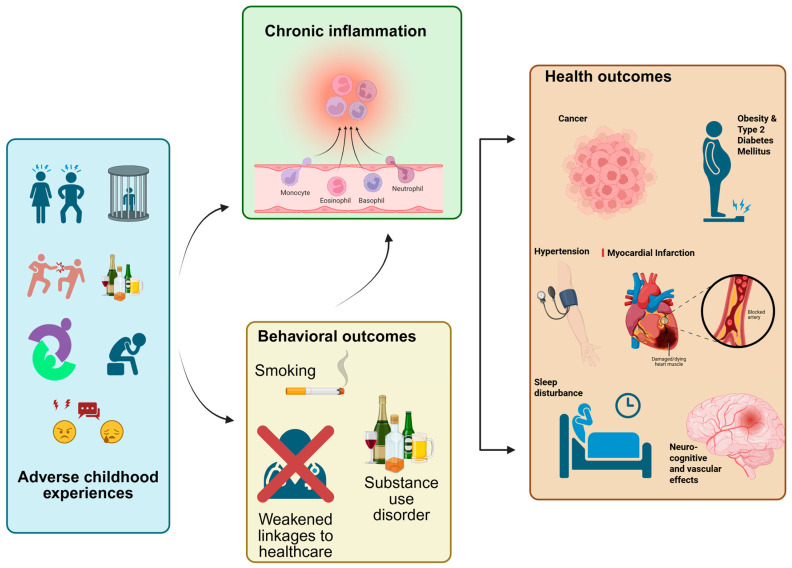
Proposed schema depicting the interactions between adverse childhood experiences with chronic inflammatory responses and behavioral responses and their long-term health outcomes.

A third mechanistic framework involves immune senescence and accelerated biologic aging. Leukocyte telomere shortening has repeatedly been associated with ACE exposure and chronic psychosocial stress [66,67]. Several studies have demonstrated shorter leukocyte telomere length among adults reporting higher cumulative childhood adversity [66,67]. Mayer et al. demonstrated that socioeconomic stressors experienced before 18 predicted shorter leukocyte telomere length and greater telomere attrition during midlife [66]. Similarly, greater cumulative ACE exposure has been associated with shorter leukocyte telomere length in otherwise healthy adults. Telomere attrition is commonly interpreted as a marker of cellular aging and immune senescence. In oncology, impaired immune fitness and T cell exhaustion have been associated with reduced antitumor immunity and diminished responsiveness to immunotherapy. In melanoma, leukocyte telomere length is associated with improved responsiveness with adoptive cellular [68]. Nevertheless, whether ACE-associated telomere shortening meaningfully contributes to immunotherapy resistance in GBM remains unknown. Existing evidence supports conceptual association between chronic stress exposure, and immune aging, but does not establish causality in neuro-oncology. Moreover, immune senescence in patients with GBM is likely multifactorial. Age, corticosteroid exposure, systemic inflammation, lymphopenia induced by chemo irradiation and tumor mediated immune suppression all contribute substantially to impaired immune function which is described in Figure 2. ACE-associated biologic aging, if relevant, would therefore likely represent only one component of a broader immunosuppressive landscape.

### 2.4. Biological Sex as a Modifier of ACE-Immunity Interactions

Sexual dimorphism in human physiology has been documented across multiple systems, including the immune system [69]. Various features of the immune system display dimorphic characteristics delineating by biological sex including immune cell composition and phenotype, immune cell homeostasis, and development [70]. For instance, skewing of lymphocytes ratios towards greater abundance of CD4 T cells and greater tissue resident dendritic cells in females are associated augmented immune function and autoimmunity [71]. In the murine brain, microglia exhibit sex-specific transcriptional profiles: transplanted female microglia reduced ischemic stroke injury in male mice, whereas transplanted male microglia did not alleviate injury in female mice [72]. These studies suggest that the immune response to a physiologic insult may display specific patterns based upon biological sex.

In the context of adverse childhood experiences, childhood adversity was associated with shorter telomere length in biological females, not biological males [68]. Parental absence has been linked to have a greater psychosocial impact on females than males [73]. Interestingly, among females, increased body mass index and frailty were associated with higher cumulative ACE score but remained stable compared to White males and was inversely correlated for Black males [74,75]. While exposure to child mistreatment is associated with higher physiological dysregulation and inflammation, the association with biological sex is more varied [75]. Inflammatory responses mediated through IL-6 showed sexually dimorphic associations with males exposed to total trauma or emotional neglect predicted higher IL-6 concentration, but not for females [76,77].

Biological sex also is associated with certain subtypes of solid tumors. In neuro-oncology, brain cancer incidence and outcomes are sexually dimorphic, with GBM showing a male-to-female incidence ratio of approximately 1.5:1 [78,79,80,81,82]. Furthermore, males tend to have worse overall survival compared to females; male sex was found to be an independent predictor of worse mortality in GBM [82]. Related to immune function, associations have been reported between lower socioeconomic status and significant impairments in immune function in men [83]. This includes reduced NK cell cytotoxicity, impaired phagocytosis, and altered cytokine production, like spontaneous cytokine release from peripheral blood mononuclear cells. modified the immune response to stress [83]. It has been shown that when male and female mice experience maternal deprivation in early life, male mice in adulthood produce significantly weaker lymphoproliferative responses to lipopolysaccharide (LPS) immune challenges compared to females, and, demonstrated significantly lower ratios of NMDAR and IL-1R subunits relative to female mice after maternal deprivation on post-natal day 9 [84,85,86,87]. Additional research is necessary to translate mechanistic findings from animal models to meaningful benefits in human studies, but together these findings support the notion that even a single early life stressor experience exerts long-term impacts on immune function. Biological sex serves as an important modifier on these differences and must be considered as the field continues to investigate how to optimize immunotherapies for treatment of brain cancer.

### 2.5. ACES and Immunotherapy

Immunotherapy is a rapidly growing field which has shown promising advances for overcoming treatment resistance, reversing T-cell tolerance and aiding in the development of cancer vaccines [88,89]. One prominent class of immunotherapies is immune checkpoint inhibitors, which modulate T cell activation through inhibitory pathways such as PD-1 and CTLA-4. Immune checkpoint inhibitors have been utilized to great success in the treatment of melanoma, with approximately 50% of patients demonstrating an objective response to combination PD-1 and CTLA-4 blockade [90]. However, a factor which continues to limit immunotherapy effectiveness is variability in patient responsiveness. Across malignancies, the proportion of patients who fail to achieve a durable response can be as high as 70%, reflecting both tumor-intrinsic and host-related determinants of immune activity [91]. In GBM, clinical trials evaluating immunotherapy have yielded largely disappointing results. For example, in a phase III trial for GBM, the addition of the rindopepimut vaccine targeting EGFRvIII to standard therapy failed to improve overall survival, highlighting the challenge of mounting an effective anti-tumor immune response in this disease [92]. Similarly, checkpoint blockade with PD-1 inhibitors has demonstrated limited efficacy in recurrent GBM, with response rates markedly lower than those observed in melanoma [40]. The Immunotherapy Response Assessment for Neuro-Oncology (iRANO) uses MRI to differentiate responders from non-responders, but imaging alone has proved insufficient to completely predict response to treatment [86,93]. Collectively, these studies underscore the substantial heterogeneity in immunotherapy outcomes among patients with GBM and highlight the need to better understand host-level modifiers of immune function that may contribute to treatment resistance.

The effectiveness of cancer immunotherapy typically hinges upon a robust response from the patient’s immune system, and it is possible that one contributing factor to variable patient responsiveness is due to our incomplete understanding of how early life stressors may lead to aberrant immune responses. As has already been discussed, ACEs have been linked to increased pro-inflammatory gene transcription and elevated peripheral levels of inflammatory cytokines [94,95,96]. Strikingly, many of these same immune parameters, particularly IL-6, T cell activation status, and myeloid cell populations, serve as predictive biomarkers of response to checkpoint inhibitor therapy. Baseline levels of circulating cytokines including interleukin-6, hepatocyte growth factor, and monocyte chemotactic protein 2 are associated with prognosis after treatment using ipilimumab and anti-PD-1 antibodies in melanoma, with patients harboring combined elevations in all three markers demonstrating a three-fold lower overall response rate compared to those without such elevations [97]. Higher baseline IL-6 has also been independently associated with shorter progression-free survival in melanoma patients receiving nivolumab plus ipilimumab [98]. Researchers have also found that response to immunotherapy is affected by both peripheral blood immune cell profile and circulating cytokine levels in lung cancer; for instance, baseline frequencies of classical monocytes, natural killer cells, and ICOS+ CD4+ T cells in peripheral blood strongly predicted pembrolizumab efficacy in advanced NSCLC [99], while pre-therapeutic elevations in IL-6 and IL-8 were associated with significantly reduced response and survival in NSCLC patients receiving checkpoint inhibitors [99]. Adverse childhood experiences (ACEs) are linked to chronic inflammation and immune system dysfunction, which can hinder the effectiveness of immunotherapy in treating diseases like cancer. High ACE scores are associated with lower IL-2 levels (vital for T-cell growth) and poorer survival rates, indicating that trauma alters the biological environment needed for successful immunotherapy [100]. Taken together, these results support significant overlap between the effects of ACEs on the immune system and parameters which are associated with efficacy of immunotherapeutic interventions.

Several immunologic markers provide prognostic insight following immunotherapy in patients diagnosed with cancer. For example, baseline levels of circulating cytokines are associated with favorable prognosis after treatment using ipilimumab and anti-PD-L1 antibodies in melanoma [101]. Researchers have also found that response to immunotherapy is affected by both peripheral blood immune cell profile^8^ and circulating cytokine levels in lung cancer [102,103]. Theoretically, dysregulation in baseline immunologic markers may represent one avenue by which ACE exposure alters immunotherapy responsiveness.

Patient responses to immunotherapy are highly variable, and this carries significant costs to patients and society. Immunotherapy carries the risk of a broad range of side effects including neurotoxicity, immunodeficiency, and autoimmunity against off-target organs which cause significant distress to the patient and may demand additional treatment [104,105]. In addition, drug prices have steadily increased in recent years, with the median launch price for cancer drugs in the United States averaging about $27,688 per month [106]. Immunotherapies including CAR T-cell therapies are particularly expensive, costing over $450,000 USD per patient, contributing to financial toxicity [107]. This is against the backdrop that these therapies are not guaranteed to work for a significant portion of patients [108]. Deepening our understanding of patient characteristics, such as history of ACEs, which can predict clinical benefit, could help guide treatment. This information could lead to superior stratification of patients into clinical trials that will maximize the benefit they receive. Thus, further elucidating the connection between ACEs and response to immunotherapy has broad implications for future clinical trial design.

More recently, two studies evaluating prospective cohorts have identified baseline emotional distress associated with reduced pathologic response and shorter median progression-free survival in patients diagnosed with melanoma and NSCLC [109,110]. These studies are significant in their approach by considering psychosocial factors like emotional distress and immunotherapy responses. ACEs are associated not only with increased cancer risk, but with anxiety and depression which can produce emotional distress [111]. Along a similar vein, other areas of medicine, such as in hematopoietic stem cell transplantation, have studied the impact of social disadvantage, socioeconomic status, and other social determinants of health categories on hematopoietic stem cell transplantation outcomes [112,113,114,115,116,117]. Like hematopoietic stem cell transplantation, immunotherapy harbors significant iatrogenic risk to patients with immense potential for treatment failure. It is imperative to include ACE indices in future studies related to brain tumor immunotherapy and to drive preclinical investigation to uncover the causal impact of ACEs on the immune system and immunotherapy responses against brain tumors. Ultimately, these efforts will aid in minimizing excess risk, limit adverse iatrogenic effects, and maximize therapeutic benefits for patients.

## 3. Limitations and Future Directions

Several important limitations should be acknowledged when considering potential relationships among ACE exposure, immune dysregulation, and GBM immunotherapy response. First, much of the literature remains correlative and derives from observation studies conducted outside of neuro-oncology. Mechanistic evidence linking ACE exposure directly to GBM development or treatment resistant is currently lacking. Many proposed pathways discussed in this commentary are extrapolated from psychoneuroimmunology, chronic stress and non-CNS oncology literature. Additionally, ACEs frequently co-exist with other psychosocial and structural determinants of health, including socioeconomic disadvantages, chronic stress, depression, anxiety, substance use, and reduced healthcare access [118]. These exposures likely exert overlapping effects, hindering isolation of the unique effects of ACEs themselves. Another limitation is the approach to measuring ACEs which relies upon instruments that utilize self-reporting, which is subject to recall bias, heterogeneity in exposure definition, and potentially confounded by impaired cognition and neurologic deficits in survivors of GBM [119]. ACE scores also compress diverse experiences into cumulative indices despite potentially distinct consequences associated with different forms of adversity [119]. In neuro-oncology, it remains unclear whether ACE related biomarkers would provide. Many markers like cytokine profiles, immune cell populations and transcriptional signatures overlap with chronic stress, infection, and other pathways. Current acute stress and lifetime stress exposure in adult life is also associated with increased inflammation and deleterious health outcomes. Future studies should evaluate if ACE-related stress differs from acute or chronic stress experienced in adult life, and currently, there is a lack of a biomarker to distinguish each other at the molecular level [120,121]. Translationally, future research should seek to integrate longitudinal psychosocial data with multidimensional immune profiling in patients undergoing immunotherapy for GBM. Studies capable of distinguishing ACE-specific effects from broader chronic or current acute stress phenotypes may help clarify whether host-level stress biology contributes meaningfully to treatment response variability.

## 4. Conclusions

In summary, ACEs are highly prevalent in the general population and are associated with poor health outcomes, including increased cancer risk in adulthood. The mechanisms behind this connection remain incompletely understood; however, growing evidence suggests that individuals with ACEs exhibit significant immune dysregulation in adulthood in the form of increased immune senescence, upregulation in inflammatory pathways, epigenetic changes, and altered cytokine secretion. These factors may compromise the immune system’s ability to prevent cancer, and ACEs may also modulate an individual’s response to immunotherapies that aim to leverage the host’s immune physiology against neoplastic cells. Other fields such as hematopoietic stem cell transplantation have studied the impact of social disadvantage and socioeconomic status on transplant outcomes, but studies involving brain tumors have lacked inclusion of ACEs and other social disadvantage indices. Emerging literature has drawn connections between baseline emotional distress and immunotherapy responsiveness, and these studies will lay a promising foundation for more comprehensive evaluations of the interplay between psychosocial factors and cancer immunotherapy. Future studies should seek to parse apart the effects of ACEs from those of anxiety and depression, given the high comorbidities of these conditions and their known associations with immune dysfunction. Understanding how past experiences impact immune function and anticancer responses will improve the future outcomes for patients diagnosed with cancer.

## Data Availability

No new data were created or analyzed in this study. Data sharing is not applicable to this article.
